# Possible Role of Phosphatidylglycerol-Activated Protein Kinase C-βII in Keratinocyte Differentiation

**DOI:** 10.2174/1874372201711010059

**Published:** 2017-10-24

**Authors:** Lakiea J. Bailey, Vivek Choudhary, Wendy B. Bollag

**Affiliations:** 1 Charlie Norwood VA Medical Center, One Freedom Way, Augusta, GA 30904, USA; 2 Department of Physiology, 1120 15th Street, Medical College of Georgia at Augusta University (formerly Georgia Regents University), Augusta, GA 30912, USA

**Keywords:** Aquaporin-3 (AQP3), Epidermis, Keratin-10, Phospholipase D2 (PLD2), Skin, Kinase

## Abstract

**Background:**

The epidermis is a continuously regenerating tissue maintained by a balance between proliferation and differentiation, with imbalances resulting in skin disease. We have previously found that in mouse keratinocytes, the lipid-metabolizing enzyme phospholipase D2 (PLD2) is associated with the aquaglyceroporin, aquaporin 3 (AQP3), an efficient transporter of glycerol. Our results also show that the functional interaction of AQP3 and PLD2 results in increased levels of phosphatidylglycerol (PG) in response to an elevated extracellular calcium level, which triggers keratinocyte differentiation. Indeed, we showed that directly applying PG can promote keratinocyte differentiation.

**Objective:**

We hypothesized that the differentiative effects of this PLD2/AQP3/PG signaling cascade, in which AQP3 mediates the transport of glycerol into keratinocytes followed by its PLD2-catalyzed conversion to PG, are mediated by protein kinase CβII (PKCβII), which contains a PG-binding domain in its carboxy-terminus. Method: To test this hypothesis we used quantitative RT-PCR, western blotting and immunocytochemistry.

**Results:**

We first verified the presence of PKCβII mRNA and protein in mouse keratinocytes. Next, we found that autophosphorylated (activated) PKCβII was redistributed upon treatment of keratinocytes with PG. In the unstimulated state phosphoPKCβII was found in the cytosol and perinuclear area; treatment with PG resulted in enhanced phosphoPKCβII localization in the perinuclear area. PG also induced translocation of phosphoPKCβII to the plasma membrane. In addition, we observed that overexpression of PKCβII enhanced calcium- and PG-induced keratinocyte differentiation without affecting calcium-inhibited keratinocyte proliferation.

**Conclusion:**

These results suggest that the PG produced by the PLD2/AQP3 signaling module may function by activating PKCβII.

## 1. INTRODUCTION

The epidermis forms the mechanical and water permeability barrier of the skin, allowing terrestrial existence and protecting from various environmental insults. The predominant cells comprising the epidermis are keratinocytes, which form a stratified epithelium. At the basement membrane, the basal keratinocytes continuously proliferate to replace damaged cells and those sloughed to the surroundings. As they move upwards into the upper epidermal layers, the keratinocytes growth arrest and differentiate, expressing different sets of genes and proteins as they become more and more differentiated. A great deal is known about the signals that regulate proliferation and differentiation, including the fact that elevated extracellular calcium concentrations trigger keratinocyte differentiation [1], Nevertheless, a complete understanding of these processes, and the signaling molecules that modulate them, requires further study.

We have previously shown that the lipid-metabolizing enzyme phospholipase D2 (PLD2) and the water and glycerol channel aquaporin-3 (AQP3) physically and functionally associate in keratinocytes to produce phosphatidylglycerol (PG) [2, 3], PG levels are increased biphasically in response to increasing concentrations of calcium, with a maximal effect at approximately 125μM [3], This dose response is similar to that reported for calcium-induced keratinocyte differentiation [4], suggesting the possibility that the PLD2/AQP3/PG signaling module might mediate keratinocyte differentiation. This idea is supported by our finding that manipulation of this module inhibited proliferation and promoted differentiation of keratinocytes [5], In particular, treatment of keratinocytes with liposomes formed from egg-derived PG promoted the differentiation and inhibited the proliferation of rapidly growing keratinocytes [5, 6], The mechanism by which PG exerted this effect, however, is unclear.

The protein kinase C (PKC) enzymes comprise a family of enzymes with 10 isoforms that are differentially regulated. The classical (or conventional) PKC isoforms, which include PKCα, PKCβI, PKCβII and PKCγ, require acidic phospholipids and are activated by increased diacylglycerol and calcium levels triggered upon phosphoinositide hydrolysis initiated by receptor engagement by various hormones, growth factors and other ligands. Different PKC isoforms are encoded by separate genes except for PKCβI and PKCβII, which represent splice variants of mRNA transcribed from a single gene; PKCβI and PKCβII differ in their C-terminal V5 regions. In PKCβII, this region is the location of the PG-binding domain and contains the molecular determinant necessary for nuclear translocation and enzyme activation [7, 8], In HL60 leukemia cells, PG in the nuclear membrane selectively stimulates PKCβII activity 3–6 fold above the level achieved in the presence of optimal concentrations of calcium, diacylglycerol and phosphatidylserine [9], In fibroblasts, entry into mitosis is dependent upon activation of PKCβII by PG [10], Furthermore, the sequence in PKCβII responsible for binding to PG has been localized to the 13 amino acids in the C-terminus unique to PKCβII [7],

We hypothesized that PKCβII might serve as an effector enzyme for PG in keratinocytes to promote early keratinocyte differentiation. We tested this idea by examining the redistribution of phospho-PKCβII in keratinocytes treated with a moderately elevated calcium concentration (which maximally increases PG levels [3]) and PG liposomes. We also assessed the effect of overexpression of PKCβII on the calcium-induced inhibition of proliferation and stimulation of keratin 10 levels. We provide evidence for PKCβII activation in response to an elevated extracellular calcium concentration and PG liposomes as well as the ability of PKCβII to promote early keratinocyte differentiation.

## 2. METHODS

### 2.1. Culture of Primary Mouse Keratinocytes

Primary murine epidermal keratinocytes were prepared from 1 to 3 day old neonatal ICR CD-1 outbred mice as described in [11]. Treatment of mice conformed to policies in the Guide for the Care and Use of Laboratory Animals and monitored by the Institutional Animal Care and Use Committee (IACUC) of Augusta University. Harvested keratinocytes were plated at a density of 25,000 cells/cm^2^ and incubated overnight at 37°C with 5% carbon dioxide in Plating Medium composed of calcium-free minimum essential medium alpha (MEMα) supplemented with 2% dialyzed fetal bovine serum, 25μM CaCl_2_, 5ng/mL epidermal growth factor, 2mM glutamine, ITS+, 100U/mL penicillin, 100μg/mL streptomycin and 0.25μg/mL fungizone as in [12], After approximately 24 hours, Plating Medium was replaced with either a laboratory-prepared serum-free keratinocyte medium (SFKM) or commercially purchased Keratinocyte-serum free medium (K-SFM) (Gibco, Gaithersburg, MD). Initial experiments used SFKM containing 25μM CaCl_2_, 90μg/mL bovine pituitary extract, ITS+, 5ng/mL epidermal growth factor, 2mM glutamine, 0.05% BSA, 100U/mL penicillin, 100μg/mL streptomycin and 0.25μg/mL fungizone as described by Griner *et al.* [12], Our laboratory subsequently switched to commercial K-SFM supplementing pre-prepared K-SFM with 50μM CaCl_2_, 2.5μg recombinant human EGF and 25mg bovine pituitary extract per the supplier’s recommendations [13], Medium was replaced every 1–2 days.

### 2.2. Preparation of PG Liposomes

Liposomes were prepared from egg-derived PG (Avanti Polar Lipids, Alabaster, AL). Briefly, PG in organic solvent was distributed into amber glass vials as 1mg aliquots, the solvent evaporated with nitrogen gas and the lipid stored under nitrogen at −20°C until use. For experiments, 0.5 mL serum-free medium was added to the amber vial to hydrate the lipid film followed by bath sonication using a Branson Sonifier with a microprobe and a cup horn.

### 2.3. RT-PCR and Quantitative RT-PCR Analysis

Cultured primary mouse keratinocytes, epidermis or skin were collected in 0.5–1mL of Trizol and RNA was extracted according to the manufacturer’s protocol. The epidermis was isolated following overnight incubation of neonatal mouse skin in 0.25% trypsin in Hank’s buffered saline solution at 4°C to allow the enzyme to permeate along the dermal-epidermal junction of the skin. After a brief incubation at 37°C to promote trypsin proteolysis, the epidermis was manually separated from the dermis using forceps. Total skin was extracted immediately after harvest. Mouse brain tissue was also collected and immediately RNA-extracted. First-strand cDNA synthesis was performed using Thermoscript RT-PCR System and oligo(dT) nucleotides (Sigma-Aldrich, St. Louis, MO) according to the manufacturer’s protocol. RT-PCR was performed using JumpStart RedTaq reaction mix (Sigma-Aldrich) and the primers for PKCβII and GAPDH (mPKCβ forward: 5’-GCTGACAAGGGCCCAGCCTC-3’; reverse: 5’-GTGTGGTTCCGTGCCGCAGAG-3’ and mGAPDH forward: 5’-GCGGCACGTCAGATCCA-3’; reverse: 5’ CATGGCCTTCCGTGTTCCCTA-3’), also according to the manufacturer’s protocol. The reaction parameters consisted of heat activation at 94°C for 2 minutes followed by 35 cycles of denaturation at 94°C for 15 seconds, annealing at 50°C for 30 seconds and elongation at 72°C for 30 seconds. The amplified product was resolved on a 1% TAE agarose gel. Quantitative RT-PCR was performed using Taqman probes (ThermoFisher Scientific, Waltham, MA) and analyzed by the delta-delta Ct method as described previously [13].

### 2.4. Western Blot Analysis

Near-confluent cultures of keratinocytes were incubated in SFKM (25μM CaCl_2_) or K-SFM (50μM CaCl_2_) alone or with medium containing the desired treatment, elevated calcium (125μM CaCl_2_) or 100μg/mL PG for 24 hours. Cells were then harvested in lysis buffer, with 30μL/cm^2^ of heated buffer (containing 0.1875M Tris-HCl, pH 8.5, 3% SDS and 1.5mM EDTA) added to each well. Protein concentrations were determined using a BioRad protein assay with BSA as the standard. After protein determination, 3X sample buffer (containing 30% glycerol, 15% β-mercaptoethanol and 1% bromophenol blue) was added to each sample to constitute Laemmli buffer. Total protein was also extracted from mouse brain, homogenized epidermis and freshly isolated keratinocytes after shearing using an 18-gauge needle. Samples were stored at −20°C until analysis at which time protein samples were heated to near boiling and equal amounts were loaded onto 8% SDS polyacrylamide gels, separated by electrophoresis and transferred to Immobilon-FL transfer membranes (Millipore, Billerica, MA). After washing and blocking, the membranes were incubated overnight with primary antibody [recognizing PKCβII (Abeam, Cambridge, MA), pPKCβII (pSer^660^, Epitomics, Burlingame, CA), 1:10,000; K10 (Covance, Denver, PA), 1:15,000; and actin (Sigma-Aldrich or Santa Cruz, Santa Cruz, CA), 1:15,000] followed by secondary AlexaFluor florescent antibodies (Invitrogen, Carlsbad, CA or Licor, Lincoln, NE, 1:10,000), all diluted in Odyssey blocking buffer containing Tween-20 (LiCor). Immunoreactive bands corresponding to the proteins of interest were visualized *via* an Odyssey®SA infrared imaging system from Li-Cor and quantified with the internal software according to the manufacturer’s instructions. The data are reported as means ± SEM after normalization to actin levels.

### 2.5. [^3^H]Thymidine Incorporation into DNA

DNA proliferation assays were performed on primary mouse keratinocytes overexpressing PKCβII or empty vector incubated in SFKM containing 25μM or 125μM calcium. Briefly, cells were transfected as previously described and exposed to experimental treatments for 24 hours. The cells were then incubated with 1μCi/mL [^3^H]thymidine (Moravek Biochemicals, Brea, CA) for 1 hour at 37°C. Reactions were terminated and macromolecules precipitated with cold trichloroacetic acid and the cells solubilized in 0.3M sodium hydroxide. [^3^H] Thymidine incorporation was measured in an aliquot using Ecolite scintillant (MP Biomedicals, Santa Ana, CA) and a Beckman Coulter LS 6500 multi-purpose scintillation counter (Brea, CA).

### 2.6. Immunocytochemistry

For immunocytochemistry PKCβII-specific and phosphoPKCβII-specific antibodies were generously provided by Dr. Denise Cooper (University of South Florida, Tampa, FL) [14]. The antibody recognizing PKCβII was raised against residues 655 to 671 (the C-terminus specific to PKCβII), and the phospho-specific antibody recognizes PKCβII phosphorylated on serine 660 of the C-terminus (residues 657–673) [14]. Primary murine keratinocytes were plated on glass BD BioCoat fibronectin-coated slides and at near-confluence were incubated in SFKM (25μM CaCl_2_) or SFKM containing 100μg/ml PG at 37°C. After the desired incubation period, cells were washed with PBS and fixed in 4% paraformaldehyde. After permeabilization in 0.2% Triton X-100, the slides were blocked in buffer containing 10% goat serum and 1% BSA in PBS and incubated in buffer containing either phospho-PKCβII (1:500) or keratin-10 (Abeam, 1:250) overnight. The slides were then incubated in Cy3-conjugated secondary goat anti-rabbit IgG antibody (1:150) in 10% goat serum at room temperature and mounted with ProLong Antifade with DAPI (Invitrogen). Staining was visualized by multiphoton microscopy with a Zeiss LSM 510 confocal laser scanning microscope with a Meta System equipped with a Coherent Mira 900 tunable Ti:Sapphire laser for multi-photon excitation at 488nm, 543nm and 760nm wavelengths (Carl Zeiss Microscopy, Germany).

### 2.7. Keratinocyte Transfection

Primary mouse keratinocytes were transfected with wild-type PKCβII plasmid in a pcDNA3 vector backbone (or the vector plasmid) *via* AMAXA nucleofection (Lonza, Cologne, Germany), using an Amaxa Nucleofector Kit for primary endothelial cells as in [15] according to the manufacturer’s instructions. The authors reported 40–60% transfection efficiency with this method [15]. The PKCβII plasmid was a generous gift from Dr. Lan Ko, (Augusta University). Transfected cells were then incubated in RPMI medium containing 10% fetal bovine serum and antibiotic/antimycotic (100U/mL penicillin, 100μg/mL streptomycin and 0.25μg/mL fungizone) for 20 minutes, plated in Plating Medium (described above) and allowed to attach overnight. After 24 hours the plating medium was replaced with K-SFM (containing 50μM CaCl_2_).

### 2.8. Statistics

All experiments were performed independently a minimum of three times. Values were analyzed for statistical significance by analysis of variance or repeated measures analysis of variance with a Student-Newmann-Keuls or Dunn’s *post-hoc* test using Prism (GraphPad Software, San Diego, CA). All quantitative data were expressed in the form of bar graphs, with the bars representing mean ± standard error ofthe mean (SEM).

## 3. RESULTS

### 3.1. PKCβII is Expressed in Mouse Keratinocytes

**Fig. (1).**
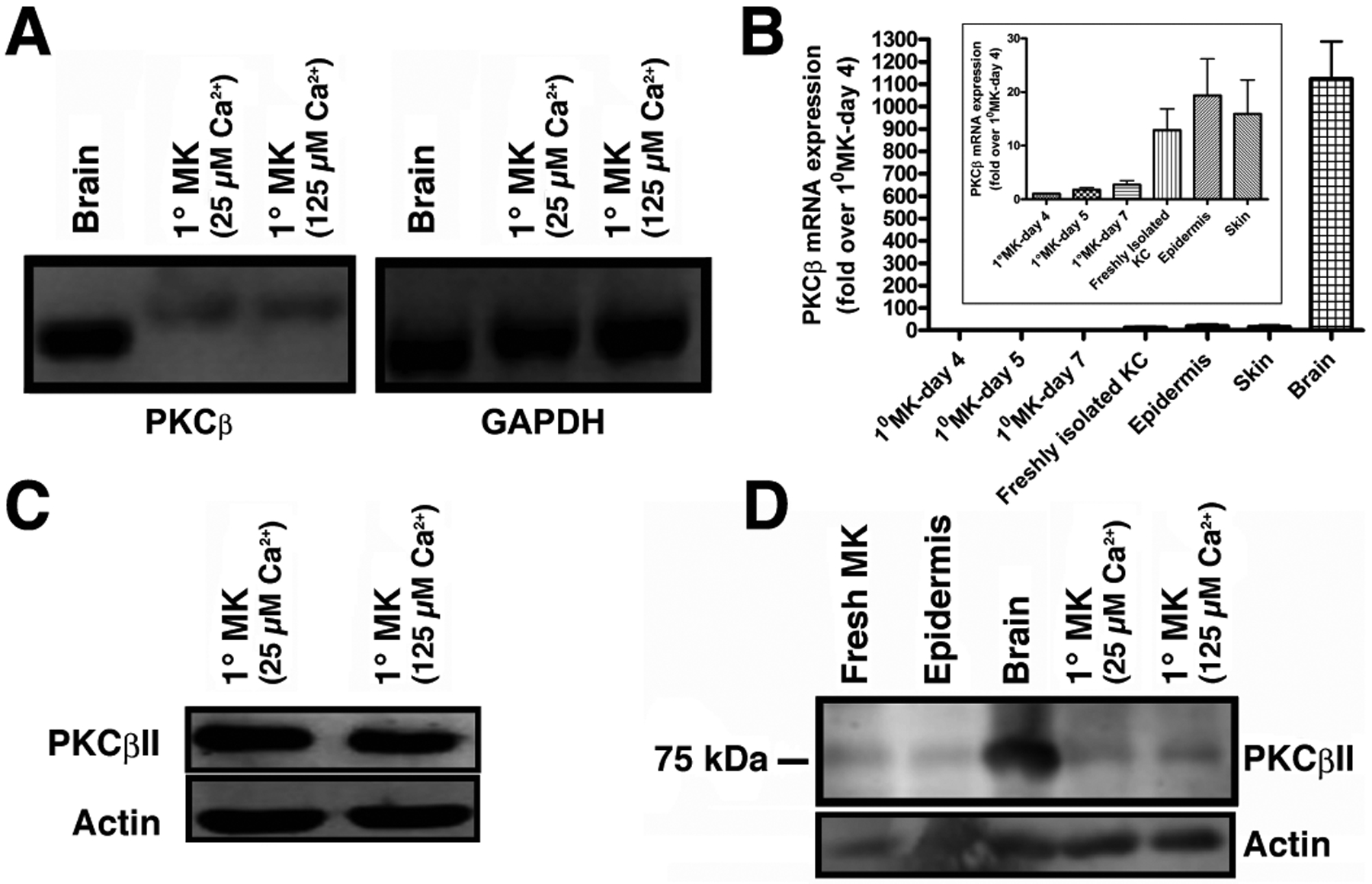
PKCβII protein is expressed in primary mouse epidermal keratinocytes, freshly isolated epidermal keratinocytes and the epidermis. **(A)** RNA was isolated from primary mouse keratinocytes (1°MK) cultured to near confluence before incubation for 24 hours in medium containing basal (25μM) or moderately elevated (125μM) extracellular Ca^2+^concentrations as indicated and PKCβ expression monitored by semi-quantitative RT-PCR. **(B)** RNA was isolated from primary mouse keratinocytes (1°MK) incubated in medium containing a basal (50μM) extracellular Ca^2+^concentration, freshly isolated keratinocytes (before plating), isolated epidermis, total skin and brain as indicated and PKCβ expression monitored by quantitative RT-PCR using primer-probe sets from Applied Biosystems and a StepOne system as described in Methods. Results are expressed as the fold change in normalized cycle threshold relative to primary cultures of primary mouse keratinocytes (1°MK) cultured for 4 days, analyzed using the ΔΔCt method with GAPDH as the normalization control. Note that the brain expresses a significantly greater amount of PKCβ than do keratinocytes or skin tissue; therefore, in the inset the values obtained only from 1°MK cultured for the indicated number of days, freshly isolated keratinocytes (KC), isolated epidermis or total skin are plotted using a different scale. **(C)** Protein lysates were prepared from primary mouse keratinocytes (1°MK) cultured in medium containing basal (25μM) or moderately elevated (125μM) extracellular Ca^2+^concentrations. **(D)** Protein lysates prepared from primary mouse keratinocytes (1°MK) cultured in medium containing basal (25μM) or 125μM Ca^2+^, freshly isolated keratinocytes (fresh MK) or a homogenate of epidermis were analyzed by western blotting. Western analysis was performed using the PKCβII antibody obtained from Dr. Denise Cooper (University of South Florida, Tampa, FL). Commercially available antibodies yielded similar results. Brain was used as a positive control.

PKCβII is known to bind to and be activated by PG to trigger cell cycle progression in human leukemia cells [8], However, although multiple PKC isozymes have been identified in keratinocytes, there has been some debate regarding the presence of PKCβ in these cells [16 – 19], with an initial report failing to detect PKCβ in mouse keratinocytes using northern analysis [16]. Two subsequent studies found PKCβ in human skin [17], mouse keratinocytes [18] and mouse skin [19]. To resolve this issue, we first sought to determine if PKCβ could be detected by semi-quantitative RT-PCR using mouse brain as a positive control. PKCβ was found to be transcribed in primary mouse keratinocytes (Fig. 1A). Although the mRNA was much less abundant than in brain (Fig. 1B), quantitative RT-PCR using Taqman assays indicated that the cycle threshold was within a detectable range (approximately 30 cycles). The difference in the amount of cDNA amplified and separated also likely explains the slight apparent differences in molecular weight of the PKCβ band observed in brain and keratinocytes with semi-quantitative RT-PCR (Fig. 1A) since greatly different amounts of nucleic acid can separate slightly differently by electrophoresis. We next sought to determine whether the PKCβII protein was expressed using a PKCβII-specific antibody obtained from Dr. Denise Cooper [14]. This antibody was raised against residues 655 to 671 (the C-terminus specific to PKCβII). Using this antibody it was shown that keratinocytes also express PKCβII protein (Fig. 1C). To ensure that expression of the enzyme was not an artifact of culture, we also demonstrated PKCβII protein expression in freshly isolated keratinocytes and epidermis (Fig. 1D), as well as mRNA expression in total skin (Fig. 1B). Therefore, we hypothesized that PKCβII might be an effector enzyme for PG in keratinocytes.

### 3.2. PKCβII is Redistributed Upon Provision of PG

**Fig. (2).**
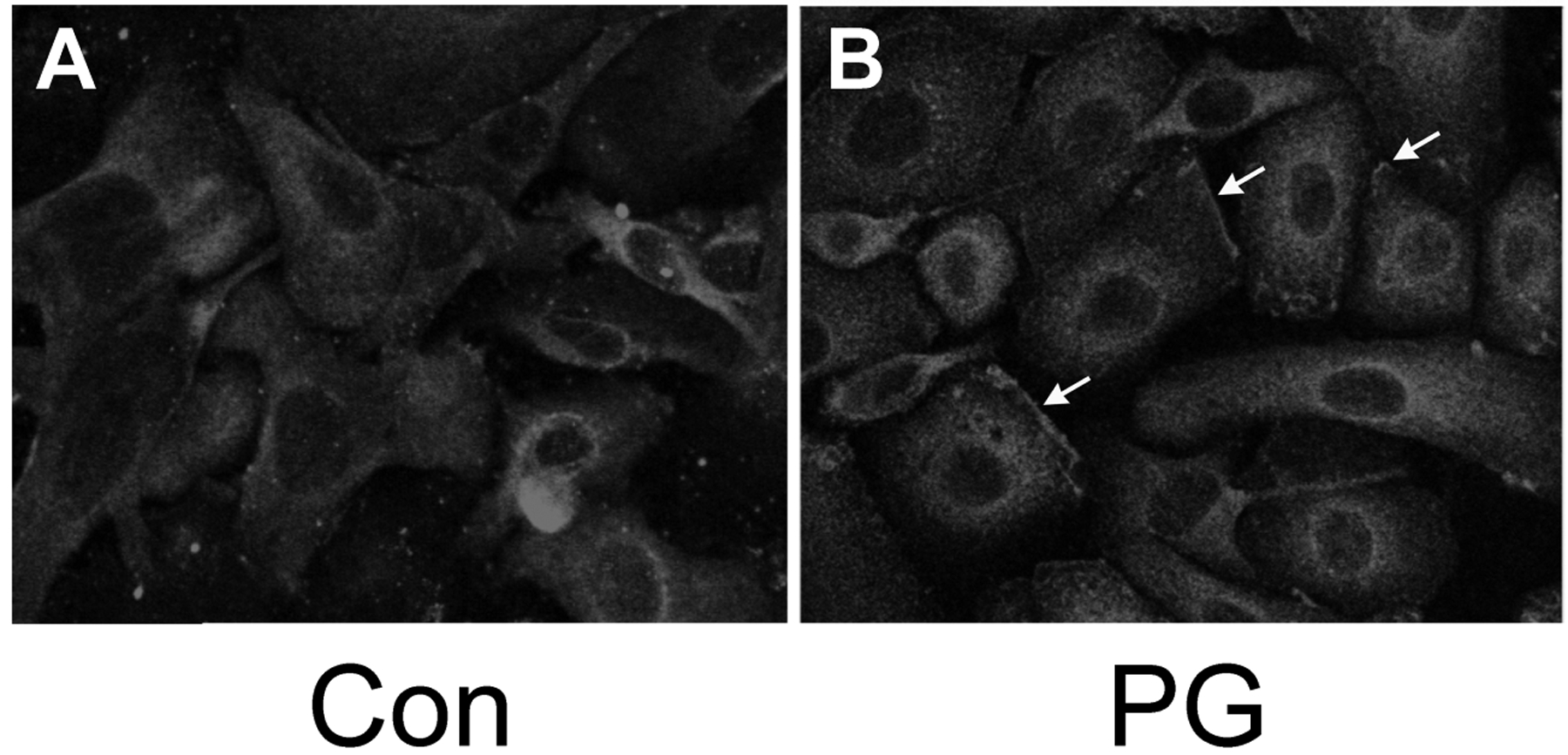
PG stimulates phosphoPKCβII redistribution in keratinocytes. Keratinocytes grown on fibronectin-coated slides were stimulated for 60 minutes with medium containing a basal (25μM) calcium concentration **(A)** without (Con) or **(B)** with 100μg/mL egg PG (PG), provided in the form of liposomes. Cells were then stained with an antibody recognizing phosphorylated PKCβII, an Alexa468-conjugated secondary antibody and the nuclear stain, DAPI, and visualized using a multiphoton Zeiss microscope. The primary antibody was omitted to serve as a negative control and showed no staining for PKCβII although DAPI was visualized (data not shown).

We have shown that PG can inhibit proliferation and promote differentiation of keratinocytes, and based on the literature demonstrating that PKCβII is a PG-activated enzyme, we hypothesized that stimulation of PKCβII activity by PG may be the mechanism by which the lipid signal exerts its effects. Phosphorylation and translocation to cell membranes are considered hallmarks of PKC activation [20], We utilized immunocytochemical techniques to visualize the cellular localization of phosphorylated/activated PKCβII upon treatment with PG, again using an antibody provided by Dr. Cooper recognizing PKCβII phosphorylated on serine 660 of the C-terminus (residues 657–673). Primary mouse keratinocytes plated on collagen-coated slides were subjected to immunohistochemical analysis. Under basal conditions, autophosphorylated PKCβII (phosphoPKCβII) was found diffusely throughout the entire cell with increased staining around the perinuclear area (Fig. 2, left panel). A 1h treatment with PG (100μg/mL egg-derived PG in the form of liposomes) increased staining in the perinuclear area (Fig. 2A, right panel) compared to the control (Fig. 2B, left panel). In addition, PG induced localization of PKCβII in the plasma membrane (Fig. 2B, right panel, arrows). Since PKCβII requires phospholipids for its activity, translocation to the membrane is thought to mark activation of the enzyme [21, 22], These results suggest that, as in leukemia cells [7 – 9], PG activates PKCβII. Treatment of keratinocytes with PG followed by western analysis with the antibody recognizing phosphoPKCβII also showed a trend towards enhanced autophosphorylation (activation) of PKCβII (to a value of 1.32 ± 0.13-fold over the control of 1.0; n=6), but the increase did not quite achieve statistical significance (p=0.054).

### 3.3. A Moderately Elevated Extracellular Calcium Concentration Induces the Autophosphorylation/ Activation of PKCβII

**Fig. (3).**
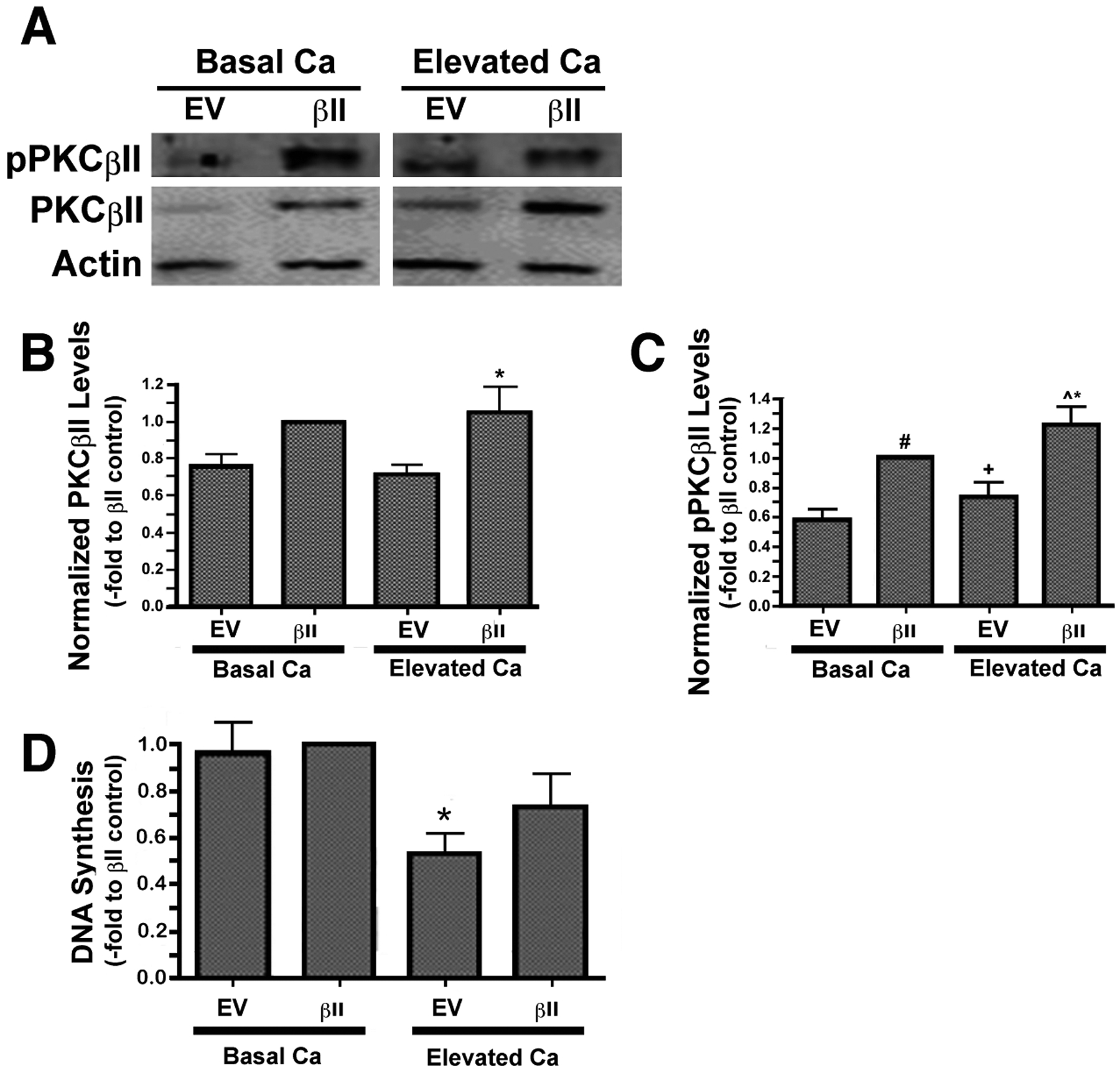
Overexpressed PKCβII is autophosphorylated/activated in response to an elevation of extracellular calcium concentration in mouse keratinocytes but has no effect on calcium-inhibited proliferation. Mouse keratinocytes were nucleofected with wild-type PKCβII (βII) or empty vector (EV) and then cultured in the presence of basal (25μM) or a moderately elevated extracellular calcium level (Ca; 125μM) for 24h. Cells were harvested and lysates were resolved on 8% SDS gels, transferred to PVDF membranes and probed with antibodies recognizing total PKCβII, autophosphorylated PKCβII and actin. **(A)** A representative experiment is illustrated. **(B)** Total PKCβII and **(C)** pPKCβII levels were quantified, normalized to actin and expressed relative to the PKCβII-transfected cells under basal conditions. Data represent the means ± SEM from at least 3 separate experiments. For total PKCβII, *p<0.05 vs EV or EV+Ca and for autophosphorylated PKCβII, #p<0.01 vs EV, *p<0.01 vs EV+Ca, ^^^p<0.001 vs EV, ^+^p<0.05 vs βII. **(D)**Keratinocytes nucleofected with wild-type PKCβII (βII) or empty vector (EV) were cultured in the presence of basal (25μM) or a moderately elevated extracellular calcium level (Ca; 125μM) for 24h. [^3^H]Thymidine was added to the medium for 1 hour and DNA synthesis measured as described in Materials and Methods. [^3^H]Thymidine incorporation into DNA is expressed as the percentage of the control value and shown as the mean ± SEM (n=4; *p<0.01 vs EV or (βII).

We have previously observed an ability of moderately elevated extracellular calcium concentrations to increase PG levels in keratinocytes [3], suggesting the possibility that PG-activated PKCβII may play a role in calcium-induced differentiation. To explore this possibility we over-expressed PKCβII and first assessed the effect of calcium on the autophosphorylation/activation of this enzyme. Primary mouse keratinocytes were transfected with either an empty vector or PKCβII plasmid vector, as described in the Materials and Methods section, and then cultured in the presence or absence of an elevated extracellular calcium concentrations. Total and autophosphorylated PKCβII levels were increased in mouse keratinocytes transfected with PKCβII plasma vector, and autophosphorylation of this overexpressed PKCβII was stimulated in response to elevated extracellular calcium (125μM) (Figs. 3A–C).

### 3.4. Over-Expression of PKCβII has no Effect on Calcium-Induced Inhibition of Proliferation but Increases the Levels of Keratin-10, a Marker of Early Keratinocyte Differentiation, in Primary Mouse Keratinocytes

To determine whether this over-expression of PKCβII affected the ability of elevated calcium levels to inhibit keratinocyte proliferation, we treated vector- and PKCβII-transfected cells with medium containing basal calcium levels or a moderately elevated calcium concentration. Proliferation was assessed by measuring the incorporation of [^3^H]thymidine into the DNA of dividing cells. In cells expressing basal, physiological levels of PKCβII, that is, transfected with empty vector plasmid, stimulation with elevated extracellular calcium resulted in calcium-induced inhibition of proliferation (Fig. 3D), as described elsewhere [23], A similar inhibition was observed in the PKCβII-transfected cells, although this effect did not achieve statistical significance, suggesting that this enzyme likely does not mediate the anti-proliferative effect of an elevated calcium concentration.

**Fig. (4).**
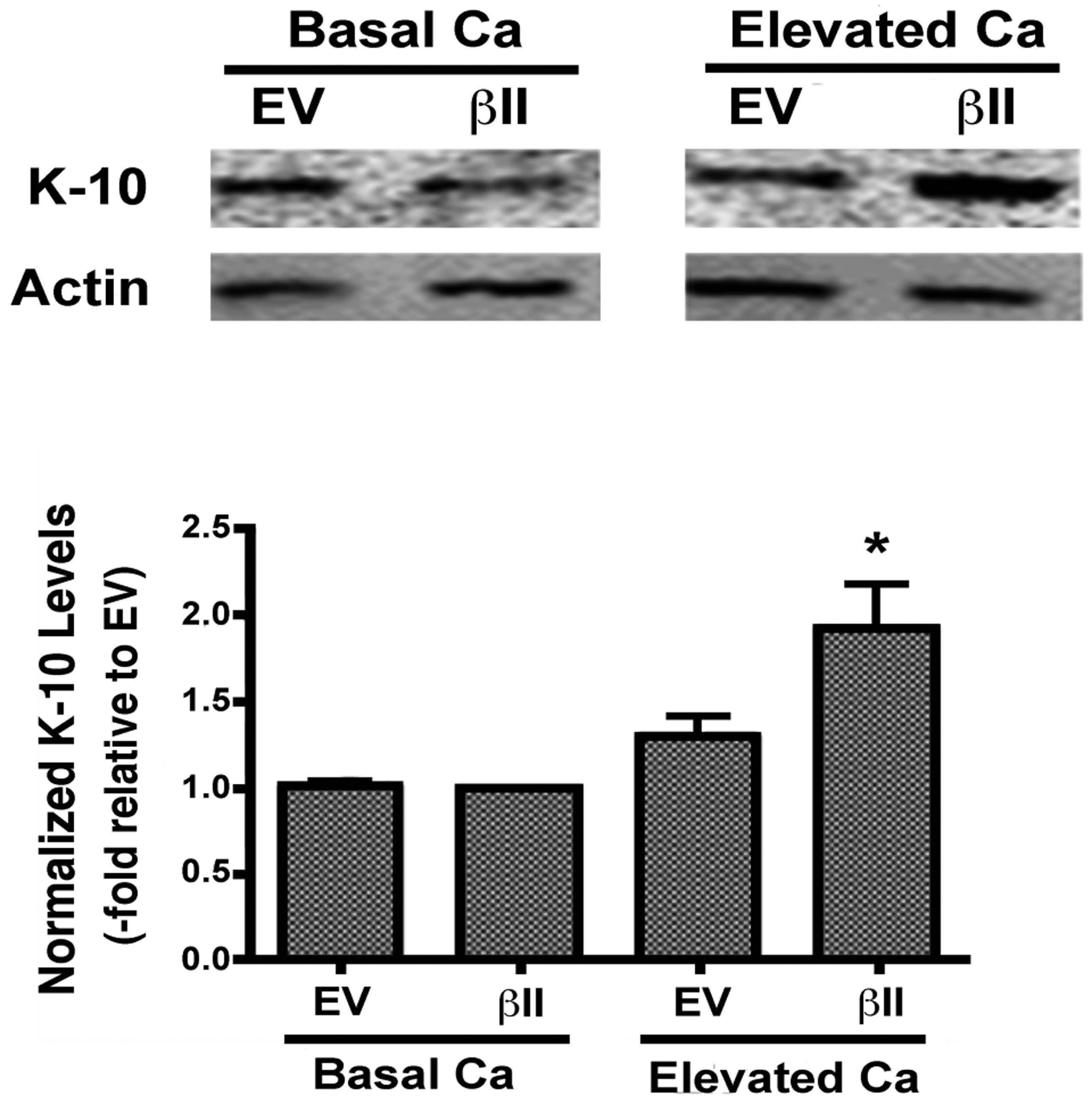
Overexpressed PKCβII enhances calcium-induced keratin-10 protein expression (differentiation). Keratinocytes nucleofected with wild-type PKCβII (βII) or empty vector (EV) were cultured in the presence of basal (25μM) or a moderately elevated extracellular calcium level (Ca; 125μM) for 24h. Following cell harvest total lysates were resolved on 8% SDS gels, transferred to PVDF membranes and probed with antibodies recognizing keratin-10 (K10) and actin. A representative experiment is shown. Keratin-10 levels (normalized to actin and expressed relative to the PKCβII-transfected cells under basal conditions) were quantified and expressed as the mean ± SEM (n=4; *p<0.01 vs EV, (βII or EV+Ca).

Expression levels of keratin-10, a marker of keratinocyte differentiation, were also examined in cells overexpressing PKCβII. There was no change in the keratin-10 expression of keratinocytes over-expressing PKCβII and cultured under basal conditions. PKCβII over-expression in combination with an elevated extracellular calcium concentration, however, resulted in a substantial up-regulation in keratin-10 expression, with p<0.01 versus all other conditions (Fig. 4). These results suggest that PKCβII alone is not sufficient to induce an increase in keratin-10 expression, but instead works in concert with calcium to promote early differentiation, but not growth arrest, in primary mouse keratinocytes.

### 3.5. Over-Expression of PKCβII Affects the Pattern of Keratin-10 Distribution and Results in an Altered Morphology of Cells Grown in the Presence of Phosphatidylglycerol

**Fig. (5).**
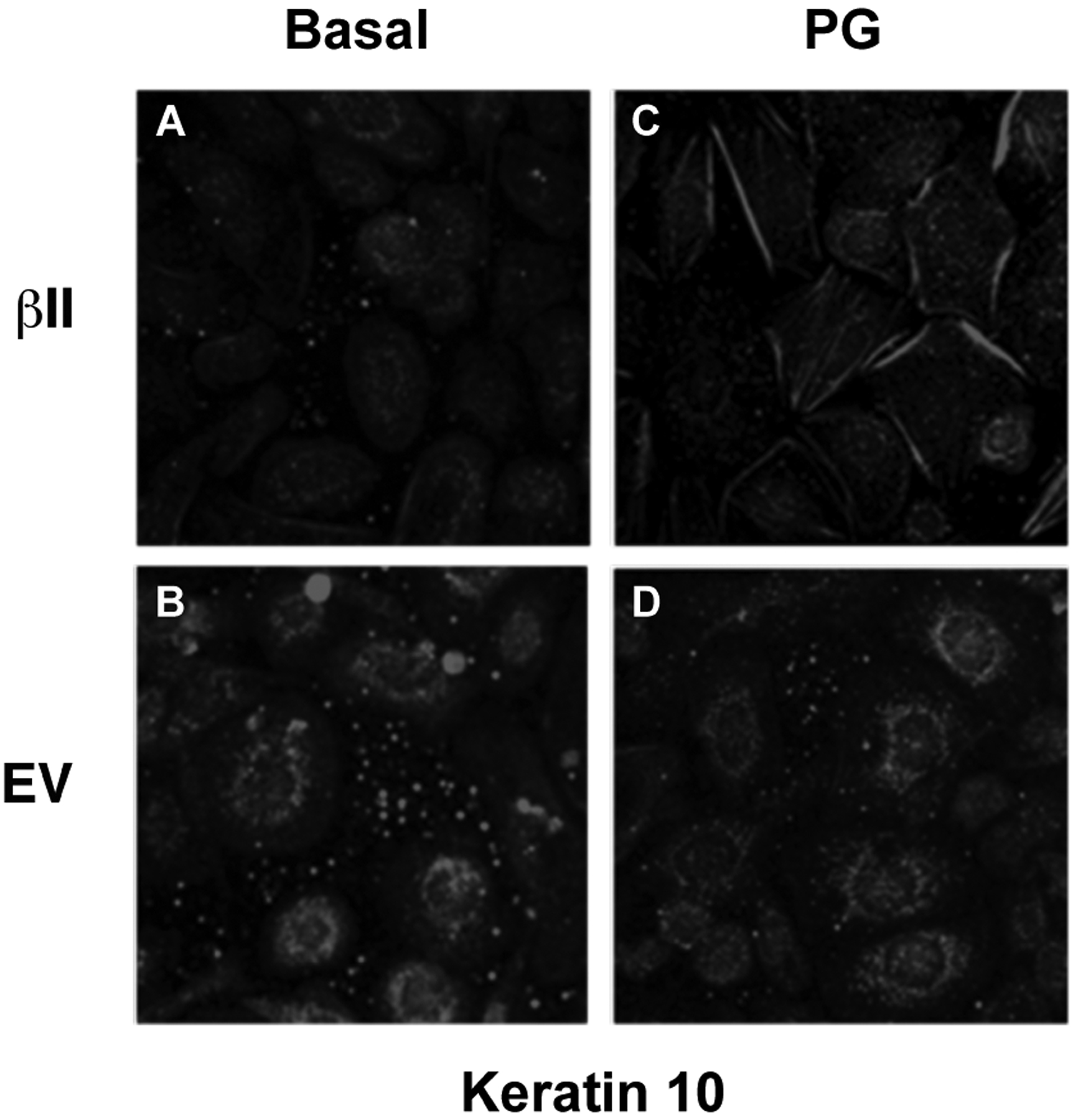
PKCβII overexpression increases the PG-induced formation of keratin-10-containing intermediate filaments. Mouse keratinocytes were nucleofected with wild-type **(A and C)** PKCβII (βII) or **(B and D)** empty vector (EV) and then cultured on coated glass slides in the **(C and D)** presence or **(A and B)** absence of 100μg/mL PG in 25μM calcium-containing medium for 24h. Cells were fixed, permeabilized, probed with an antibody recognizing keratin-10 and visualized with an Alexa468-conjugated secondary antibody. Immunofluorescence was examined by confocal microscopy.

Our previous findings suggested that PG stimulates keratinocyte differentiation and inhibits proliferation [5, 6]; therefore, we next examined the morphological effect of PG on keratinocytes over-expressing PKCβII. Mouse keratinocytes were again transfected with either PKCβII or empty vector and were then cultured on collagen-coated slides in medium containing basal calcium with or without 100μg/ml egg PG. The cells were then fixed, permeabilized and stained with an antibody specific for keratin-10 (Fig. 5). Interestingly, treatment with PG led to morphological changes consistent with later differentiation in cells transfected with PKCβII (Fig. 5A–D), as well as the formation of keratin-10 filaments, changes that were not seen in cells transfected with empty vector and stimulated with PG (Fig. 5D). Thus, PG in PKCβII-overexpressing cells induced enlargement and flattening reminiscent of the alterations observed with later keratinocyte differentiation; these changes also were not seen in keratinocytes overexpressing PKCβII in the absence of PG (Fig. 5A). These data suggest that keratinocyte differentiation in response to elevated calcium concentrations may be mediated through the activation of PKCβII induced by increased production of PG. This hypothesis is consistent with the observed ability of PKCβII overexpression to increase keratin-10 levels in the presence of a moderately elevated calcium concentration and of PG to promote differentiative changes in PKCβII-overexpressing keratinocytes.

## 4. DISCUSSION

Disruption in the normal form and function of skin can result in a significant amount of human suffering. Several human skin diseases, such as psoriasis, a hyperproliferative disorder of the epidermis, and the non-melanoma skin cancers (basal and squamous cell carcinoma) are the result of a breakdown in the carefully controlled program regulating the proliferation and differentiation of keratinocytes. Approximately 7 million Americans and as much as 3 percent of the world population suffer from the devastating effects of psoriasis (wwww.healthline.com/health/psoriasis/facts-statistics-infographic). Although usually not a fatal condition, the physical and emotional impact of psoriasis has been reported to be comparable with that of other serious medical conditions, including heart and lung disease, depression and cancer ([24, 25] and www.aad.org/media/stats/conditions/psoriasis). Basal and squamous cell carcinomas are the two most common skin cancers in the world, with more than 3.5 million new diagnoses each year in the United States (http://www.cancer.org). Our results contribute to the body of knowledge regarding the pathways regulating the normal growth and differentiation of epidermal keratinocytes that are dysregulated in these diseases. Our study provides insight into a possible role of PKCβII in keratinocyte differentiation. On the other hand, an inhibitor of PKC has been proposed as therapeutic option for psoriasis, based on its ability to reduce cytokine production in psoriatic patients [26], Our results suggest that targeting a PKC inhibitor more towards PKCθ and PKCα, and less towards PKCβ, might improve the efficacy of such a therapy (although PKCα has also been demonstrated to play a role in keratinocyte differentiation [27]).

Although the precise mechanisms regulating the progression of keratinocytes through the multilayered stratified structure of the epidermis remain unknown, our laboratory has proposed a potential signaling module involving PG generation by a signaling module composed of AQP3 and PLD2. We have previously shown a functional and physical interaction between PLD2 and the glycerol channel AQP3 [2, 3]. Notably, the PLD2/AQP3 signaling module was observed to be abnormal in psoriasis and non-melanoma skin cancers [28], suggesting a possible involvement of dysregulation of this module in such hyperproliferative skin diseases. Our laboratory has further shown that: (1) PLD2 can utilize AQP3-transported glycerol to generate PG, (2) elevated calcium concentrations increase PG levels and (3) this increase is likely mediated by PLD [3]. Maximal stimulation of calcium-induced PG formation was observed at a calcium concentration optimal for stimulation of markers of early differentiation (*e.g*., keratin-10) [3, 4], These findings, combined with the observation that the C-terminal PKCβII V5 region binds PG and contains the molecular determinant necessary for translocation and activation of the enzyme [7, 8], led us to suspect that PKCβII may be the mediator of PG’s ability to promote keratinocyte maturation. Here, we present evidence that PKCβII is present in mouse keratinocytes (Fig. 1), consistent with previous reports [18, 19]. Although the brain expresses significantly more PKCβ than do keratinocytes, keratinocytes express PKCβ mRNA, although the band migrated slightly differently than the amplicon from brain, likely because of the difference in amounts in the two tissues, since abundance can alter electrophoretic separation. Nevertheless, PKCβ mRNA was also demonstrated by quantitative RT-PCR using Taqman assays. Since the Taqman probe only binds to the specific amplicon of interest, artifactual amplification of incorrect sequences will not be detected. In addition, we noted that mRNA levels tended to increase under conditions when keratinocytes would be expected to show less stem cell character and greater differentiation. This idea likely explains why PKCβ expression tends to rise with increasing time in culture as the cells reach confluence and begin to undergo contact-induced differentiation [29] and also to be higher in freshly isolated keratinocytes, which contain differentiated cells that do not attach to the tissue culture plastic upon seeding for culture. Expression tends to be higher also in epidermis and skin, as these tissues also contain large numbers of differentiated keratinocytes. The presence of PKCβ has also been detected in human skin [17]; nevertheless, a dearth of PKCβ isoform-specific antibodies with reactivity in formalin-fixed, paraffin-embedded tissue samples has hampered a complete characterization of PKCβII protein expression in human skin.

We also found that PKCβII is translocated/activated in response to PG treatment (Fig. 2). In addition, PKCβII over-expression induces an up-regulation of keratin-10 upon calcium-induced stimulation of differentiation (Fig. 4), but has no effect on calcium-induced inhibition of proliferation (Fig. 3). Finally, overexpression of PKCβII in keratinocytes promotes keratin-10 filament formation and results in morphology consistent with later differentiation upon treatment with PG (Fig. 5). On the other hand, it could be argued that a toxic effect of the liposomal matrix is responsible for the reorganization of keratin filaments observed in (Fig. 5), since the concentration of egg PG used, 100 μg/mL, translates to approximately 120–130 μM, near the threshold for toxicity observed by Mayhew *et al*. [30], Nevertheless, overall cell morphology was not markedly affected by PG as seen in (Fig. 2), suggesting that toxicity is likely not an issue.

Calcium functions as a precise regulator of keratinocyte maturation and is essential for normal differentiation [4, 31 – 33], such that an increase in extracellular calcium concentration can initiate this process. Thus, keratinocytes grown in a low-calcium medium proliferate and maintain an immature state *in vitro*, but will transition to a more differentiated state when exposed to elevated extracellular calcium levels [34, 35], Consistent with this effect, a calcium gradient has been observed in the epidermis *in situ*, with the lowest concentration observed in the basal layer where keratinocytes are actively proliferating and gradually increasing outward towards the more differentiated granular layer [33], The observation that extracellular calcium is able to increase PG levels led us to test whether PKCβII plays a role in calcium-induced differentiation. Thus, we experimentally altered the expression of PKCβII and recorded the effect of this manipulation on keratinocyte proliferation and differentiation. Proliferation was assessed by measuring the incorporation of [^3^H]thymidine into DNA in cells transfected with vector or PKCβII. In cells expressing basal, physiological levels of PKCβII (*i.e*., vector-transfected cells), stimulation with elevated extracellular calcium resulted in calcium-induced inhibition of proliferation (Fig. 3), as described by Bikle and colleagues [23], However, overexpression of PKCβII did not substantially alter this calcium-elicited inhibition, suggesting that PKCβII is not involved in the initial growth arrest triggered by calcium.

In an effort to determine if PKCβII plays a role in keratinocyte differentiation we next evaluated the effect of PKCβII overexpression on the levels of keratin-10, a marker of early differentiation, in the presence and absence of elevated extracellular calcium. Although we did not detect a statistically significant change in the levels of keratin-10 under basal conditions, PKCβII overexpression in combination with elevated extracellular calcium resulted in a substantial up-regulation of keratin-10 levels (Fig. 4). These results suggest that PKCβII alone is not sufficient to induce an increase in keratin-10 expression, but instead works in concert with calcium to promote early differentiation in primary mouse keratinocytes.

These results suggest that PKCβII can be activated not only by PG, but also by agents, such as extracellular calcium, that stimulate keratinocyte differentiation. This result, as well as our previous data indicating that PG can induce keratinocyte differentiation [5], prompted us to test the morphological effect of PG on keratinocytes overexpressing PKCβII. Interestingly, treatment with PG led to morphological changes consistent with entry into later differentiation in cells transfected with PKCβII. This morphological change was not detected in keratinocytes that were expressing basal levels of PKCβII (Fig. 5), suggesting that overexpressed PKCβII must be activated (by PG or the PG produced upon elevation of extracellular calcium levels) in order to exert its prodifferentiative effect.

## CONCLUSION

In summary, we show that PKCβII is present in mouse keratinocytes and is translocated and/or activated upon stimulation of the AQP3/PLD2/PG signaling module by a moderate elevation of extracellular calcium levels (which increases PG levels [3]) or direct provision of PG. We provide further evidence suggesting that PKCβII, activated in response to an elevated calcium level or provision of PG, can promote keratinocyte differentiation. This result suggests a potential mechanism by which PG affects keratinocyte function.

## LIST OF ABBREVIATIONS

AQP3: Aquaporin-3
K10: Keratin-10
K-SFM: Keratinocyte serum-free medium
PG: Phosphatidylglycerol
PKC: Protein kinase C
PKCβII: Protein kinase C-betaII
PLD2: Phospholipase D2
SFKM: Serum-free keratinocyte medium
